# An integration engineering framework for machine learning in healthcare

**DOI:** 10.3389/fdgth.2022.932411

**Published:** 2022-08-04

**Authors:** Azadeh Assadi, Peter C. Laussen, Andrew J. Goodwin, Sebastian Goodfellow, William Dixon, Robert W. Greer, Anusha Jegatheeswaran, Devin Singh, Melissa McCradden, Sara N. Gallant, Anna Goldenberg, Danny Eytan, Mjaye L. Mazwi

**Affiliations:** ^1^ Department of Critical Care Medicine, Hospital for Sick Children, Toronto, ON, Canada; ^2^ Institute of Biomaterials and Biomedical Engineering, Department of Engineering and Applied Sciences, University of Toronto, Toronto, ON, Canada; ^3^ Institute of Medical Sciences, University of Toronto, Toronto, ON, Canada; ^4^ Executive Vice President for Health Affairs, Boston Children’s Hospital, Boston, MA, United States; ^5^ School of Biomedical Engineering, University of Sydney, Sydney, NSW, Australia; ^6^ Department of Civil and Mineral Engineering, Faculty of Applied Science and Engineering, University of Toronto, Toronto, ON, Canada; ^7^ Department of Surgery, Division of Paediatric Cardiac Surgery, Hospital for Sick Children, Toronto, ON, Canada; ^8^ Translational Medicine, Peter Gilgan Centre for Research & Learning, Toronto, ON, Canada; ^9^ Department of Emergency Medicine, The Hospital for Sick Children, Toronto, ON, Canada; ^10^ Department of Bioethics, The Hospital for Sick Children, Toronto, ON, Canada; ^11^ Division of Clinical and Public Health, Dalla Lana School of Public Health, Toronto, ON, Canada; ^12^ Genetics & Genome Biology, Peter Gilgan Centre for Research & Learning, Toronto, ON, Canada; ^13^ Department of Computer Science, University of Toronto, Toronto, ON, Canada; ^14^ Vector institute for Artificial Intelligence, University of Toronto, Toronto, ON, Canada; ^15^ CIFAR, Toronto, ON, Canada; ^16^ Department of Medicine, Technion, Haifa, Israel; ^17^ Department of Pediatric Critical Care, Rambam Medical Center, Haifa, Israel

**Keywords:** Integration engineering, artificial intelligence, machine learning, digital health, system of systems (SoS), human factors engineering (HFE), healthcare (MeSH)

## Abstract

**Background and Objectives:**

Machine Learning offers opportunities to improve patient outcomes, team performance, and reduce healthcare costs. Yet only a small fraction of all Machine Learning models for health care have been successfully integrated into the clinical space. There are no current guidelines for clinical model integration, leading to waste, unnecessary costs, patient harm, and decreases in efficiency when improperly implemented. Systems engineering is widely used in industry to achieve an integrated *system of systems* through an interprofessional collaborative approach to system design, development, and integration. We propose a framework based on systems engineering to guide the development and integration of Machine Learning models in healthcare.

**Methods:**

Applied systems engineering, software engineering and health care Machine Learning software development practices were reviewed and critically appraised to establish an understanding of limitations and challenges within these domains. Principles of systems engineering were used to develop solutions to address the identified problems. The framework was then harmonized with the Machine Learning software development process to create a systems engineering-based Machine Learning software development approach in the healthcare domain.

**Results:**

We present an integration framework for healthcare Artificial Intelligence that considers the entirety of this *system of systems*. Our proposed framework utilizes a combined software and integration engineering approach and consists of four phases: (1) Inception, (2) Preparation, (3) Development, and (4) Integration. During each phase, we present specific elements for consideration in each of the three domains of integration: *The Human, The Technical System,* and *The Environment.* There are also elements that are considered in the interactions between these domains.

**Conclusion:**

Clinical models are technical systems that need to be integrated into the existing *system of systems* in health care. A systems engineering approach to integration ensures appropriate elements are considered at each stage of model design to facilitate model integration. Our proposed framework is based on principles of systems engineering and can serve as a guide for model development, increasing the likelihood of successful Machine Learning translation and integration.

## Glossary of key terms used in this manuscript

Artificial Intelligence (AI): A field that combines computer science and robust datasets to solve problems. These systems can be said to think like humans, act like humans, think rationally, or act rationally (1).

Food and Drug Administration (FDA): Regulatory body of the government responsible for maintaining health and safety of humans and animals within the United States through regulating food, drug, and technology (2).

Human Factors Engineering (HFE): A field of engineering focusing on the design of technology tailored to people as well as sociotechnical integration involving large, complex systems such as healthcare (3, 4).

ISO/IEC/IEEE: Global product, technological, procedural, and engineering standards set by globally recognized ISO/IEC/IEEE organizations (5, 6).

Machine Learning (ML): A branch of artificial intelligence and computer science that describes the ability of an algorithm to “learn” by finding patterns in large datasets (7).

Software Development Lifecycles (SDLC): The various software development frameworks that are used to structure software development (8).

System of Systems (SOS): A system that is composed of other systems and its “elements are managerially and/or operationally independent” (9).

## Introduction

Artificial Intelligence offers transformational opportunities in medicine, but this potential remains limited by a translation gap (10, 11). There are a variety of drivers that contribute to this gap. First, restrictions in data sharing limit training, validation and improvement of models (12). Second, lack of data and model transparency limit clinicians’ ability to interpret the model and evaluate it for relevance, accuracy and bias impacting their trust in the model and thereby limiting utilization (12–14). Third, the absence of established model verification processes impose further challenges (12–14). Financial constraints, limited physician training in the field and rules and regulations that often lag technological advances further affect model integration (12–14). While the Food and Drug Administration has proposed guidelines to regulate some clinical models (15, 16), there are no current guidelines for clinical model integration. The term “model integration” is a more appropriate term than “implementation” as it recognizes that Artificial Intelligence models need to be compatible with the complex sociotechnical environments that characterize healthcare. Integration is defined as “an act or instance of combining into an integral whole” and refers to combining several implemented elements to form a fully realized system that enables interoperability between the various elements of the system (9). Improper integration of new systems may lead to additional costs, patient harm, damage to other systems, and decrease in efficiency (9). To address this translation gap, we present a systems engineering framework to guide the development of models with explicit consideration of elements that are crucial for successful model integration in healthcare.

## Foundations

### Systems engineering

**Figure 1:**
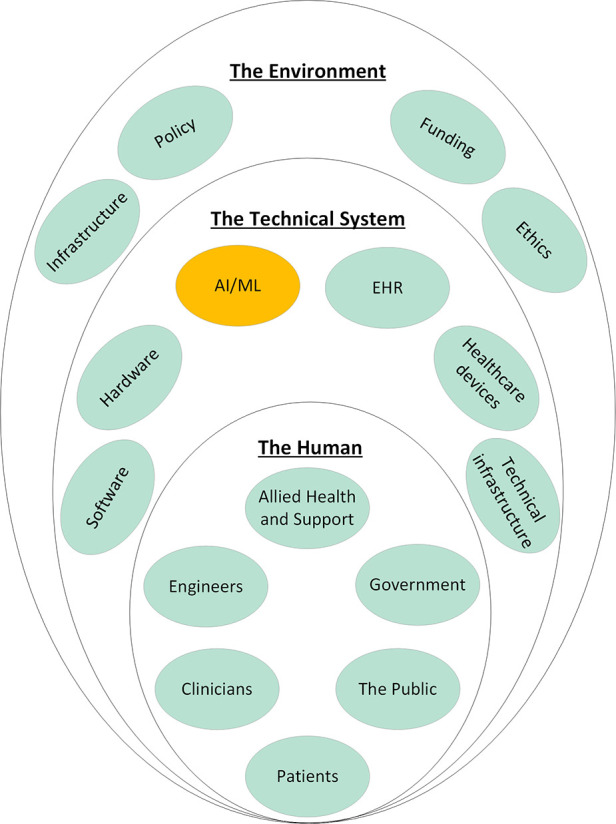
System of systems of healthcare and how applied machine learning should integrate into these existing systems. Each of the elements shown here influence every other element in an interconnected network. Electronic Health Record (EHR); Artificial Intelligence/Machine Learning (AI/ML).

Systems engineering is an interdisciplinary approach to system design to ensure its interactive elements are organized to achieve the purpose of the system (17, 18). The term dates to the early 1940's and Bell Telephone Laboratories where it was used during World War II (19). The need for systems engineering came from the discovery that satisfactory components did not necessarily combine to produce a satisfactory system (20). This was particularly a problem for industries which produced complex systems at an early date, such as communications and aircraft industries (20). Systems engineering forms the foundations of ISO/IEC/IEEE and is currently applied in a wide variety of industries from manufacturing to engineering and aerospace (18). A system is defined as “*an aggregation of elements organized in some structure to accomplish system goals and objectives, is usually composed of **humans and machines** and has a definable structure and organization with external boundaries that separate it from elements outside the system* (4).” A Machine Learning model should really be considered as a system composed of the model and data sources, its users, and its context. At higher degrees of abstraction, a system can be composed of other systems to create a system of systems (SOS), defined as a system whose “*elements are managerially and/or operationally independent”* (9). The healthcare system is a SOS (Figure 1) that results from the integration of the medical system, the regulatory, legal, and ethical systems, the financial system, the hospital system, the electronic health records, and many others. Therefore, for applied Machine Learning to become part of this existing SOS, it must be effectively integrated. Integration is the key to viability of any SOS (21) and achieving it requires effective collaboration between these inter-operable systems (9). Integration, therefore, is a process that is analyzed, planned, designed, developed, executed, managed, and monitored throughout the system's entire lifecycle and not just a distinct phase at the end (9).

Integration should consist of integrating the technical aspects of the system as well the human-system aspects. Technical integration of systems ensures that the various technical aspects of the system can work together to achieve the common objective (9). In the context of applied Machine Learning, this requires the interoperability of the models with the existing hardware and software infrastructure. To facilitate the technical integration of software, modular programming styles as well as Software Development Life Cycle (SDLC) frameworks have mechanisms that require developers to understand the existing systems and evaluate how the new system fits within this existing system as they progress through iterative development cycles. This is particularly important in dynamic industries like healthcare. The human-system integration aspect, also known as the sociotechnical integration, refers to the integration of a system within the social construct of the environment (9). Social demands as well as the societal or cultural values can play a major role in determining optimal performance of the entire system (22). Sociotechnical integration can be achieved through Human Factors Engineering (HFE), which is a field of engineering focusing on the design of technology tailored to people as well as sociotechnical integration involving large, complex systems such as healthcare (3, 4). The Systems Engineering Initiative for Patient Safety model provides a framework for integrating HFE in to healthcare to improve patient care quality and safety (23). Currently in its third iteration, this framework strongly advocates for a human centered design approach to engineering various aspects of the health care system such that the needs of patient and the people who care for these patients are put at the center of the design process (23).

Within systems engineering, the domains of integration are *The Technical System, The Human,* and *The Environment* and the interactions between them which have been previously described in literature (24) and summarized in Table 1. While this framework for integration has been used in the industry, it has not been applied to integrating Machine Learning in healthcare.

**Table 1:** Domains of integration and the interaction between them.

Domains of Integration	Definition
The Technical System	“An aggregation of elements organized in some structure to accomplish system goals and objectives, is usually composed of humans and machines and has a definable structure and organization with external boundaries that separate it from elements outside the system” (4)
Human	“An individual, a group of individuals, or organizations which have connections to the system in the form of owners, users, operators, managers, service providers, supplies, producers, or other stakeholders, who directly or indirectly have an interest in the system.” (9)
Environment	“All the relevant parameters that can influence or be influenced by the system in any lifecycle phase.” (9)
System-Environment interaction	A physical interaction occurs through technical interfaces while a non-physical interaction can occur through laws, regulations, policy, market demands and political interests, which may influence or be influenced by the system (9).
Human-System interaction	The physical, logical, or emotional relationship between the human and the system that can be influenced by or influence the system. HFE largely aims to optimize this interaction (9, 25).
Human-Environment interaction	Relationship between the human and the internal and external workplace or system environment. Some examples include organizational attributes that may affect decision-making processes of humans, circumstances that may cause deviation from standard operating procedures, impact of noise, temperature, illness, fatigue, interpersonal relationships, etc. can also influence the system or be influenced by it (9, 25). HFE can also be used here to optimize some of these challenges.

HFE, Human Factors Engineering.

### Systems engineering in software development

Systems engineering principles are applied in software development. The SDLC defines the development process of software (8). It also relates to the architecture of the software and facilitates an understanding of the required resources for the software (8). The use of agile software development techniques has facilitated software implementation but is seldom used by healthcare Machine Learning model developers. This approach also fails to recognize some of the unique implications of applied Machine Learning in practice. The purpose of SDLC has evolved over its 60-year history from ensuring an understanding of what needs to be done to focusing on structured development methods, to focusing on product delivery (26). To achieve this, there needs to be a balance between the structured and agile SDLC frameworks (26). The evolution of these different life cycles has been in response to the increasing complexity of software, the systems for which new software are being designed, advancements in hardware, and the widespread use of software in society. Despite successful use of software globally, a strong emphasis on time to market has led to the incomplete application of many well-known SDLC recommendations, particularly those of requirement gathering, planning, specifications, architecture, design, and documentation (27). There are also inherent limitations to each of the SDLC models that can further contribute to poor software design (Table 2). In addition to cost, technical concerns, need for workflow alterations, privacy concerns, perceived lack of usefulness, productivity loss, and usability issues have contributed to the very slow uptake of healthcare software such as electronic health record systems in United States (30).

**Table 2:** Overview of current software development life cycle models and their limitations (8, 28, 29).

SDLC	Overview	Limitations
Classical Waterfall model	- Series of processes in succession without gap - Foresees defect or fault - Requires proper planning and well-articulated documentation - “Characterize before the design” - Used in safety critical systems where phases and processes are inter-dependent and there is a high need for assurance with no tolerance for mistakes	- Not flexible - Prototypes made late in the overall process - Product delivery often delayed - High risk and uncertainty - Not suitable for complex and object-oriented projects - Not suitable for long and ongoing projects - Not suitable for existing systems
Iterative Waterfall model	- Use iterations to prototype and refine the project's requirements before proceeding with the waterfall model for the rest of the development process	- Iteration is possible but predisposed to errors and costly - Not suitable for long term projects - Difficult to gather requirements - Changes in previous stages can cause big issues in subsequent stages
Prototyping model	- Leverage the use of prototypes to clarify and refine requirements - May use prototype to iteratively build the finished project or simply use it as a demonstration of what is being proposed as a solution	- Requires system modifications after implementation - Can increase complexity of the system - Leads to incomplete applications
Evolutionary model	- Requirements change over time and the initial design evolves with user interaction and input as well as with new requirements	- Planning and design phase are incomplete - Not suitable for incremental building - Costly
Spiral model	- Combination of top-down and bottom-up constructs - Can be used with other models - Breaks a project into smaller segments so simplify development and evaluation - For systems when cost and risk assessment are key - Also, when users are uncertain about their needs	- Costly - Requires high expertise for risk analysis - Risk analysis central to project success - Not suitable for small projects
V-model	- Focus on validation and verification—the product from each phase is checked and approved before moving on to the next phase	- Very rigid - Prototypes are available late in the development phase - Changes require lots of documentation
RAD model	- Rapid, iterative design of small parts of the project to put into test and ensure project on track and meeting requirements before pursuing the next iteration - Agile software development falls in this category	- Depends on strong member performance to identify requirements - Only suitable for modular systems - Requires very skilled developers with good modeling skills - Costly

SCLD, Software Development Life Cycle; RAD, Rapid Application Development.

### Challenges of machine learning and existing software development life cycle

There are multiple frameworks proposed for Machine Learning development that essentially focus on *context understanding, data curation, data modeling,* and *production and monitoring* (31–34)*.* Yet, despite relatively fast and cheap development and deployment of Machine Learning models, they have been difficult and expensive to maintain and integrate (35). This is in part due to existing challenges with medical software and in part due to some unique Machine Learning issues (35). Table 3 illustrates some of the Machine Learning challenges that impact its development.

**Table 3:** Summary of challenges associated with machine learning life cycle (35).

Aspects of software Development	Features and challenges with Machine Learning development
Software Requirements	- Uncertain requirements (conceptual description of the goal after applying Machine Learning systems; different data and different application context would lead to different requirements) [M1] - Quantitative measures such as accuracy define requirements with little regard to functional requirements (the exact desired quantitative measures (e.g., accuracy) are not always known) [M2] - Requirement validation requires a larger number of preliminary experiments, ideally with real data [M3] - Requirement must consider the predictable degradation in performance of Machine Learning systems (must be degradation-sensitive and adapt to degradation through ongoing training or re-training) [M4]
Software Design	- Insufficient emphasis on the coupling of components (e.g., quality of data processing and performance of Machine Learning models) [M5] - Flexible detailed design with need for multiple, iterative experimentation to develop an effective model [M6]
Software Construction and Tools	- Bulk of coding is focused on developing an effective Machine Learning model [M7] - Debugging focused on improving model performance (need real data and often delayed until last stages) [M8] - Debugging can take a very long time based on data size and complexity of a model [M9] - Bugs can be hidden in the data [M10]
Software Testing and Quality	- Hard to reproduce test results because of sources of randomness [M11] - Testing output are often a range or probability based rather than a single value [M12] - Quality of testing is highly dependent on the quality of the test case and testing dataset [M13] - Good testing results cannot guarantee performance in production or generalizability (highly dependent on similarities of training/testing datasets and the real-world data) [M14]
Software Maintenance and Configuration Management	- Expect performance degradation [M15] - Require configuration management to keep track of varying models and associated tradeoffs, algorithm choice, architecture, data, hyperparameters, etc. [M16]
Software engineering Process and Management	- Overestimation of what Machine Learning can do leading to mismatch of expectation and reality [M17] - Limited incorporation of domain expertise into the engineering and management process [M18] - Sustained performance requires ongoing monitoring and planned evaluation to determine timing to retrain and to rectify mistakes and unexpected consequences [M19] - No standard guidance for the management of Machine Learning development [M20]

ML, Machine Learning.

### Challenges with machine learning model integration into healthcare

Developing Machine Learning models for healthcare imposes unique challenges that can impact successful clinical integration. Some challenges relate to the social complexity of medicine and others to the safety critical nature of medical systems. Table 4 summarizes some of the described challenges and gaps in clinical Artificial Intelligence models and the health care environment that limit their use. For example, health care data can be very noisy and as such, often subject to data preprocessing. This preprocessing may result in training and testing data that may not be representative of the “real world” data that the model will experience in practice (Table 3, M14; Table 4, C7). Variations in institutional Electronic Health Record and the information in the structure of this type of data also challenge model performance across institutions (45).

**Table 4:** Challenges with machine learning models in healthcare.

Aspects of Machine Learning-models and the healthcare environment	Gaps or Challenges
Context	- Need to thoroughly understand the clinical data being used for model development (13, 36) [C1] - Need models with impactful clinical utility (13) [C2] - Need models that fit within the environment they are intended for (13, 37–41) [C3]
Data	- Need access and availability to well labeled, high quality, large datasets (13, 14, 39) [C4] - Need consistency in data collection techniques (13) [C5] - Need to acknowledge and minimize inaccurate or incomplete data (13, 41) [C6] - Need to ensure that model training/test data is representative of what the model will experience during operation; consider pre-processing of data and its effect [C7] - Need to identify, remove, and account for biased data (13, 14, 40, 41) [C8] - Need to account for data shifts and their effect on model performance [C9]
Model validation and performance	- Need to conduct and develop clinical validation studies (11, 13, 14, 37) [C10] - Need to conduct clinical impact/outcome studies as Machine Learning metrics (accuracy, precision, etc.) often do not map directly to clinical performance indicators (14, 37) [C11] - Need model transparency (11, 39, 41) [C12]
Ethics and Regulation	- Need regulation and safe use guidelines (14, 39, 42) [C13] - Need privacy and cybersecurity regulations (39–41, 43) [C14] - Need to screen for algorithmic biases (11) [C15]
Financial issues	- Need adequate resources (hardware, expertise, software, etc. all in high demand, limited, and expensive) to develop and integrate models (39) [C16]
Knowledge gap	- Need users to have sufficient knowledge to interpret model output or compare different models (11, 39, 41, 44) [C17]

ML, Machine Learning.

## Methods

A modified narrative review method was used to understand (a) the challenges and gaps in integrating Machine Learning models in health care, (b) challenges associated with Machine learning models and current SDLC, and (c) principles of integration engineering from a systems engineering and human factors engineering perspective. A narrative review method is the searching of the literature with a specific goal in mind where manuscripts are hand-selected for inclusion based on the research questions (46). The ACM-DL, PubMed, and IEEE Xplore databases were used for this narrative review with the following search terms: (“All Metadata”:“machine learning” OR “All Metadata”:“artificial intelligence” OR “All Metadata”:“algorithm” OR “All Metadata”:“model”), (“All Metadata”:“healthcare” OR “All Metadata”:“medicine” OR “All Metadata”:“clinical” OR “All Metadata”:“health care” OR “All Metadata”:“health”), (“All Metadata”:framework), (“All Metadata”:development), and (“All Metadata”:“software development lifecycle”). The search was restricted to publications in the last 5 years and original peer-reviewed research and reviews. Duplicate results were removed based on manuscript title, and relevant manuscripts were selected based on abstracts. The selected manuscripts were then reviewed, summarized, and synthesized to outline (a) challenges and gaps in integrating Machine Learning Models in health care, (b) challenges associated with Machine learning models and current SDLC, and (c) principles of integration engineering from a systems engineering and human factors engineering perspective.

To address some of the challenges associated with Machine Learning models and current SDLC as well as Machine Learning models for the health care environment, we adapted existing software development guidelines as well as principles of systems engineering to develop our framework described below. The framework was iteratively designed through synthesis of the literature with expert input from the research team in domains of human factors engineering, Machine Learning, medicine, and software development. Consensus was achieved on the final iteration of the framework which was organized in keeping with steps in Machine Learning model development. In the supplementary material, we apply this framework to the design of an arrhythmia detection model intended for clinical integration.

## Results

**Figure 2:**
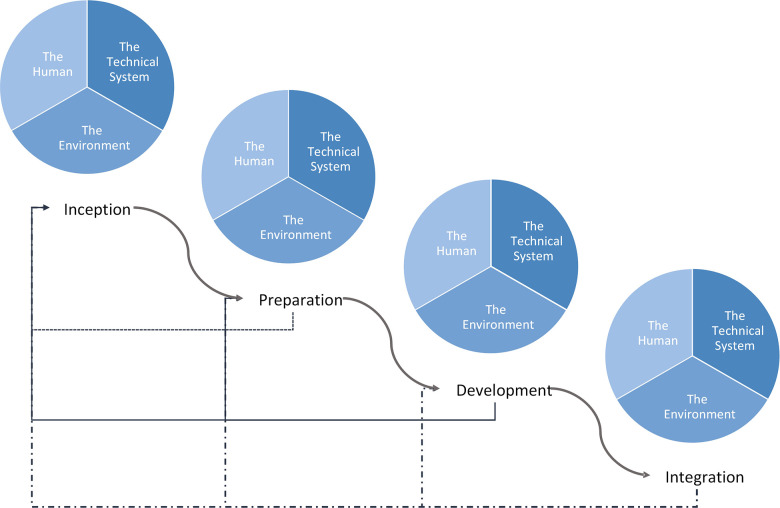
Proposed healthcare AI integration framework. Curved arrows show the progression through the integrated AI development process while the straight arrows show feedback from each phase into a previous phase. The framework begins in the top left with Inception and moves down and to the right, culminating in Integration.

We present a generalizable framework (Figure 2) that identifies four phases in the development of Artificial Intelligence models in healthcare: (1) Inception, (2) Preparation, (3) Development, and (4) Integration. Each phase incorporates considerations from the key domains of integration and systems engineering as well as the interaction between them for an integrated SOS as we show below. Outcomes from each phase while informing the phase that follows, also provides feedback to previous phases, particularly when there are new findings in a phase that were not previously considered. The challenges outlined in Table 3 that are addressed with our proposed model are indicated in [M#] format while challenges from Table 4 are indicated in [C#] as the features and steps are described. We have applied this framework to an arrhythmia detection model and have implemented this as a best practice at the Hospital for Sick Children. This practical application is demonstrated in the supplementary material. It is important to note, that the successful integration of a system into an existing system also requires ongoing maintenance and refinement which includes detection of performance degradation, changing workflows and policies, changing hardware and data acquisition, and changing knowledge and familiarity with Artificial Intelligence [M15, M19, M20]. This maintenance phase is not included in our integration framework as this follows successful initial model integration which is our focus.

### Inception

Inception refers to the very first phase of model development during which an appropriate use case and modelling approach are identified. During this phase, specific considerations for the *Technical System* include clear problem definition and clinical applications [M1, C1 & C2], strategies, and techniques to address the problem [M1], the kinds of data the model would need and whether the data is available or can be made available [M5, M13 & M14], sourcing the data, and whether the data considered for training is similar to what would be used in the intended environment [M5, M13, M14, C1-4, C7]. Close collaboration between clinicians (the domain experts and future users), model developers, and data scientists is essential. The optimal way of achieving this collaboration is through the expertise of project managers, product owners, and business analysts who specialize in gathering requirements, documenting specifications, uncovering pain points, defining business key performance indicators [M1-4, M16, M18, C1-3, C10-11, C14, C16, C17]. Given the need for a wide variety of expertise, close collaboration of a multidisciplinary team is central to the successful design and development of Artificial Intelligence models for healthcare. Our proposed inception phase incorporates integration considerations into the *context understanding* phase of current Machine Learning model development lifecycles.

*The Human* considerations during this phase include identification of all stakeholders (clinicians, data scientists, and modeling experts) and clear problem definition [M17, M18, C1 & C2]. As part of defining the problem, it is important to identify existing human challenges with the problem (e.g., who is affected by the problem, how and why), any previous attempts at addressing the problem (e.g., what has been tried, what has worked, what failed and why), and ensuring a Machine Learning solution is suitable to the problem. Some potential strategies to achieve these objectives at this phase include stakeholder engagement, informal focus groups, and immersion in the problem space. Early involvement of stakeholders provides them with a greater understanding of the limitations of Machine Learning as well as investment in model evaluation, knowledge translation, integration, and monitoring (4, 25). Similarly, Machine Learning model developers and data scientists can also gain insight into the nature of the medical problem they are solving, as well as the unique features and properties of the data they would analyze and the environment they would be designing for.

Some *Environmental* considerations during this phase include an understanding of environmental constraints, the kinds of necessary hardware for the model training and operation, and data storage. This assessment of the environment can allow for more accurate estimates of cost as it relates to the clinical integration of the model which can facilitate estimations on feasibility of integration as well as future evaluation of balancing measures and project costs [C16]. Supplementary Table S1 illustrates the practical application of this phase of our proposed framework as it relates to the arrhythmia detection model.

### Preparation

The *Technical System* related considerations during the preparation phase ensure relevant data are consistently, accurately, and reliably acquired and labelled for model training and evaluation [C4-7]. This is particularly important in clinical data as high-quality labels require the expertise of clinicians who are often restricted by availability. In addition, disagreements between multiple clinicians labeling the data can also introduce noise to the data and impact model training [C4-5, C7]. Preliminary analysis is also done on the “real” data to ensure it is suitable for the model as imagined [M3, M5, C1, C7] as well as ensuring any systematic bias or inaccuracies are identified and addressed [C8, C15]. This phase combines the current model development lifecycle phases of *data curation, data modeling* while also incorporating integration considerations.

*Human* considerations include the completion of a formal needs assessment, as well as cognitive and workflow analysis to identify specific needs and inefficiencies [M1, C2, C3]. User requirements are further defined and clarified in this phase through engagement with representatives from all stakeholders [M1, M18]. This can be achieved through cognitive task analysis, task analysis, workflow analysis, focus groups, interviews, and simulations. This establishes expectations which are calibrated during model development. Knowledge gaps among model users should be formally studied and identified to guide the development of a knowledge translation plan in the Development phase [C17]. This includes knowledge gaps in the clinical field for which a model is being designed for.

*Environmental* considerations in this phase include an evaluation of existing privacy and data security measures, as well as ethical and policy regulations that may need to be further developed to facilitate model integration and clinical utility. Supplementary Table S2 illustrates the practical application of this phase of our proposed framework as it relates to the arrhythmia detection model.

### Development

During this phase, the *Technical System* (the model and its associated user interface) is developed through iterative design, testing, and statistical evaluations. This process should mimic that of agile software design (RAD model) with rapid modeling and testing of the model output evaluated against clinical gold standards and with real production datasets [M6, M7, M14, C7 & C11]. Other SDLC models can be more expensive, slower to iterate and develop, and present fewer opportunities for user and stakeholder engagement throughout the development cycles. This rapid and iterative development and testing would allow early and real-time feedback on the performance of the model [M8-10, M13, & M17] and generate performance matrices that can be used in testing with users to determine acceptable ranges of performance based on the clinical context of the application [M2, & M12]. The results of these assessments and the features of the model at each stage should be recorded to allow future auditing as well as the establishment of a track record and rigor that would be important in establishing clinician trust [M16]. Model output should also be reproducible through thoughtful selection of training and testing data instead of random sampling [M11]. These objectives can be realized through iterative retrospective studies as well as prospective silent trials that can be coupled with simulation testing to evaluate some of the *system-human* interaction considerations. If explainable Artificial Intelligence is required to achieve transparency of a model and facilitate its clinical integration, the explanations should also be developed during this time and evaluated together with the technical system itself for both accuracy and relevance, as well as usability and impact on decision making as described below [C12, C16]. Other means of achieving transparency include ensuring data properties (including pre-processing techniques), algorithmic properties, validation testing results, and other properties are well documented and disclosed to users as needed [C12]. These would be most relevant at the initial adoption phase of a model until its performance is experientially understood by users. The *production* phase of current model development lifecycles correlates to this new proposed phase.

The technical system is also refined based on considerations of the *Human* which involve a variety of formative and summative assessments that ensure user centered design and a robust knowledge translation strategy. Simulations and other human factors assessments should be used during this phase to evaluate the model's fit within the cognitive schema of users and their existing or proposed workflow. These investigations allow for further improvements to the model. Based on the stage of the model development, simulations of varying fidelity, from low fidelity tabletop activities to evaluate workflow and the model's user interface, to high fidelity in person simulations done in near-live environments with potentially real or realistic patient data and scenario can be conducted. Another opportune moment to understand relevant interactions is during silent trials where the model is run on real data, but its output is not made visible to the clinicians providing direct patient care. At the same time, the model can be made available to off-service clinician representatives from the previously defined user groups, and their interactions with the tool as well as its effect on their decision making, workflow, efficiency, accuracy, team dynamics, and much more can be studied and evaluated [C3]. The results of such a study can be fed back to the system developers to further optimize the system as well as used by policy makers and HFEs to refine the environment, training materials, education sessions, policies and much more. Depending on the complexity of the system and the fidelity of the simulation, these studies should be repeated until there is consistency and satisfaction in the performance of the SOS. It's important to note that through these simulations, broader user engagement can be achieved which would serve as a medium through which the stakeholders are made familiar with a tool that is under development as well as the ways in which it is being proposed for use clinically [M17-19, & C16].

The *Environmental* considerations during this phase ensure the development and security of software and hardware that facilitate optimal model operation within its intended environment. Infrastructure is also evaluated and optimized for various failure modes, inefficiencies, and instabilities. Necessary policies, regulations, and rules of engagement with the model, as well as ethical and privacy guidelines would also be developed and evaluated during this phase of model development [C13, C14]. Supplementary Table S3 illustrates the practical application of this phase of our proposed framework as it relates to the arrhythmia detection model.

### Integration

The *Technical System* at this phase should be performing optimally for its intended problem, stakeholders, and healthcare context. During this phase, the model is launched for a prospective clinical evaluation [C10] and further refined based on live performance and user feedback [M19]. These prospective clinical evaluations should be subject to the peer review process to facilitate model adoption [C10]. Prospective studies should also be conducted to evaluate any long-term effects of model adoption (e.g., performance degradation secondary to practice changes) [C10] as well as any indications to suspect algorithmic biases [C14]. Finally, system monitoring should be ensued. This includes monitoring system metrics (e.g., server load, throughput, latency, etc.), input metrics (e.g., number of missing values, failed event detections, minimums, maximums, means, standard deviations, central frequencies, etc.), and output metrices (e.g., null predictions, model confusion, rate of change, etc.). It should also be evaluated for possible effects of data shift secondary to practice driven changes in data which may be due to the use of the model itself [M15, C9].

The *Human* considerations involve the launch of the developed knowledge translation plan prior to model deployment with ongoing just-in-time training when the model is clinically deployed. User engagement in the previous stages to develop knowledge translation strategies as well as evaluate model performance and usability would have allowed opportunities to calibrate user expectations to model capabilities and performance. Nevertheless, a formal knowledge translation process before the launch of the technical system and during the integration phase will further calibrate these expectations and establish a functional understanding of the technical system's operation and place within the existing workflow [M1, M17, C17]. Ongoing cost, workflow, and cognitive assessments are also leveraged as a feedback mechanism to further refine the overall system [C2].

The *Environmental* considerations during this phase, in addition to rolling out the developed and tested infrastructure as well as policies, procedures, privacy, and ethical considerations should also include an ongoing evaluation and refinement of these in practice. Supplementary Table S4 illustrates the practical application of this phase of our proposed framework as it relates to the arrhythmia detection model.

Upon the completion of this last phase, the Artificial Intelligence system is expected to be fully integrated into its intended healthcare environment and ready for clinical use.

## Discussion

The healthcare environment is a complex SOS with multiple integrated systems from a wide variety of domains. Recognizing this complexity, our framework takes a systems engineering approach to Machine Learning model design for integration.

The interdisciplinary approach promoted by systems engineering ensures that the interactive components of a system are organized to achieve the purpose of the system (9, 10). For Machine Learning system design in the healthcare space, utilizing clinical, legal, ethical, and human factors expertise is as important as ensuring adequate Machine Learning, data science, and infrastructure expertise in the design and development process. Therefore, these aspects of a healthcare Machine Learning system are incorporated from inception and the involvement of experts from these different disciplines are expected throughout the development lifecycle of such systems in our framework. Involving project managers and business analysts, for example, can further strengthen the collaboration among different domain experts and facilitate the development of a well-designed product. End-user engagement is also emphasized in our framework as early and close engagement with the development of Machine Learning systems for healthcare will lead to improvements in system performance and fit. Greater transparency from this early and collaborative engagement, as well as user driven development of model explanations contribute to end-users' trust and adoption of Machine Learning.

As the number of integrated models for the healthcare domain increases and as users become increasingly familiar with Machine Learning models in healthcare, the need to integrate models designed in other clinical environments and institutions will increase. Our proposed framework could be used to evaluate these existing models for their fit into the new SOS. Issues with performance due to differences in data properties, fit in the workflow, usability, policies, environmental space, hardware requirements and others can be identified through the steps in this framework. Solutions to these issues can then be developed and tested until deemed adequate before the model is integrated into the new SOS.

Finally, the transformational opportunities that Machine Learning, and more broadly Artificial Intelligence, offer the field of medicine and healthcare, range from improving quality and efficiency in healthcare, improving accessibility, personalizing medicine, to advancing the field of medicine and healthcare. These models are fundamentally different to the current technology used in healthcare and they can not only move research in medicine from a hypothesis driven model to one that is data driven, but also modify clinical decision-making to be more data driven as well (47). The rigorous development and integration of these models is therefore crucial in maximizing their beneficial impact on healthcare.

## Limitations

The framework that is proposed here aims at guiding the development of models for healthcare from their inception to their integration into the intended clinical space. For systems to be successful, they should also have a maintenance plan which would allow for ongoing modifications to optimize the system within its broader SOS in response to rare scenarios that may not have been encountered during the development phase as well as changes in the SOS that occur over time. For Machine Learning models, this is particularly relevant as the introduction of new technologies and changing practice patterns can have significant implications for the data based on which these models operate, potentially leading to a deterioration in model performance. The degradations in model performance should be continuously monitored and acted upon to ensure ongoing acceptable performance and reliability in their clinical application. To ensure due attention to integration without overlooking the important features in integration and maintenance, the detailed description of a maintenance phase for health care models was left out of this manuscript. We intend on exploring and developing a comprehensive maintenance phase in our future work. This will include guidelines on monitoring and maintaining longitudinal key performance indicators and model performance, as well as establishing thresholds for model retraining, workflow modifications, user re-training, and environmental modification (e.g., new policies, technology, and laws).

## Conclusion

Artificial Intelligence models in healthcare are technical systems that need to be integrated into an existing system of systems that also includes the human and the environment. An integration engineering approach allows the creation of a pragmatic framework that we believe will both address the translation gap and inform and support regulatory approaches to Artificial Intelligence models in healthcare.

## Data Availability

The original contributions presented in the study are included in the article/Supplementary Material, further inquiries can be directed to the corresponding author/s.
