# Supplementary material for: An integration engineering framework for machine learning in healthcare
Source: Front Digit Health. 2022 Aug 4;4:932411. doi: 10.3389/fdgth.2022.932411 (PMC9386122; doi:10.3389/fdgth.2022.932411)
Supplement: Supplementary file 1 [file Table_1.pdf]

Supplementary Materials

Supplementary Table 1 | Practical application of the proposed framework during the ***Inception*** phase

**Framework**	**Arrhythmia detection**
**Technical System**	
1. What is the problem Artificial Intelligence is being used to address?	*1. Early and accurate detection of arrhythmias in pediatric patients in the Cardiac Intensive Care Unit. Will focus on detecting Junctional Ectopic Tachycardia first before expanding to the detection of other arrhythmias.*
2. Why is the problem being addressed with Artificial Intelligence?	*2. Arrhythmia detection in pediatric Cardiac Intensive Care Unit patients is a difficult task and requires additional training in the field to diagnose accurately and rapidly. Junctional Ectopic Tachycardia is a common arrhythmia experienced by many post-cardiac surgery patients in the Cardiac Intensive Care Unit and it can be hemodynamically destabilizing if not recognized promptly and managed correctly. Given that access to expertise in arrhythmia detection may be limited or delayed for any given instance of Junctional Ectopic Tachycardia in the Cardiac Intensive Care Unit, a sociotechnological solution that could facilitate the diagnosis and management of Junctional Ectopic Tachycardia would be of clinical benefit to patients in the Cardiac Intensive Care Unit.*
3. What kinds of techniques could be used to build the model and why?	*3. Electrocardiogram heart rhythm classification has been widely studied in the literature resulting in multiple commercial products (Apple Watch and AliveCor). Previous arrhythmia detection models have used both classic feature engineering and deep learning approaches with good success*(1–4) *and a recent paper by Hannun et al. (2019)*(1) *demonstrated that the sensitivity of their ML model exceeded the average cardiologist sensitivity for a broad range of distinct arrhythmias from single-lead Electrocardiograms. We have experimented with several neural network architectures (VGG, Inception, EfficientNet, and ResNet). To date, the most performant model architecture is a WaveNet variant we developed for the 2020 PhysioNet Challenge*(4).
4. How will the model address the problem?	*4. The model should detect the type of arrhythmia (in this case, detect Junctional Ectopic Tachycardia) and notify the clinician of this potential diagnosis. It would be nice if it also provided a series of recommended actions to definitively diagnose the arrhythmia and to treat it.*
5. What kinds of data would the model need?	*5. Would need: Electrocardiogram waveforms, central venous pressure and arterial blood pressure waveform, appropriate arrhythmia labels to create a variety of labeled data sets, patient date of birth, admitted date, time, and bedspace in the Cardiac Intensive Care Unit. Nice to have data: the type of cardiac lesion and cardiac surgery*
6. Is the data needed for model development, validation, and function available?	*6. The Cardiac Intensive Care Unit where the model is being developed for stores all patient physiologic data, including waveforms, as well as demographic and admission data in a database called AtriumDB*(5)*. Data from this database can be accessed and utilized to develop this proposed model. Data from AtriumDB will be used for model training, validation, and function, allowing for model development on realistic data.*

**Human**	
1. Who are the stakeholders in this project? Have they been engaged in this project?	1. *Cardiac Intensive Care Unit staff physicians, medical trainees, nurse practitioners (NP) and nurses (RN). The electrophysiology staff and medical trainees. Patients and their parents, the chief informatics officer, chief medical officer, chief nursing officer, chief of research, bioethicists, human factors engineers, model developers, data scientists, and biomedical engineering. All stakeholders have been engaged in various capacities and support this project.*
2. Who would use the model and how?	2. *Cardiac Intensive Care Unit staff physicians, medical trainees, NPs, and RNs as well as Electrophysiology staff physicians and trainees are the intended clinical users of this model. Technically, the model should also be usable to those who are maintaining the model and ensuring its smooth operation as well as researchers.*
3. Are the relevant users represented in the study team?	3. *The study team consists of two staff intensivists, one NP with previous experience as a RN in the same Cardiac Intensive Care Unit and expertise in human factors engineering, one Electrophysiology staff physician and a senior medical trainee in Electrophysiology, one Cardiac Intensive Care Unit medical trainee, two data scientists, three machine learning experts, and a network administrator.*
4. Is the problem being pursued one that is clinically relevant?	4. *The problem of arrhythmia detection was initially identified by the clinicians in this Cardiac Intensive Care Unit and similarly acknowledged by the Electrophysiology experts.*
5. What are the measures of impact and how can they be measured?	5. *Improvements in accurately diagnosing Junctional Ectopic Tachycardia as well as greater efficiency in its diagnosis as measured by time to diagnosis and time to obtaining the appropriate diagnostic Electrocardiogram to confirm. Also measure, incidence of false positives and the time required to clinically evaluate and reject them, incidence of false negatives, time to initiation of appropriate Junctional Ectopic Tachycardia therapy and duration of time spent in hemodynamic instability while in Junctional Ectopic Tachycardia. Also evaluate perceived efficiency of Junctional Ectopic Tachycardia diagnosis and treatment by staff intensivists, Electrophysiology specialists, medical trainees, NPs, and RNs. While the optimal impact measure is patient outcome as measured by mortality, length of stay, and morbidity to name a few, as these are difficult to assess without a large, multi-centre trial, these acceptable surrogates are employed for impact assessment.*
**Environment**	
1. Where is the model intended for?	1. *The model is intended for the Cardiac Intensive Care Unit*
2. What are the necessary hardware and software to train and deploy the model?	2. *To train the model, we will leverage the existing Clinical Deep-Learning Infrastructure to access the required data and computational hardware required. Specifically, we will leverage the GPU (Graphics Processing Unit) capacity, AtriumDB for data access, and Jupyter notebooks for programming. Additionally, Github and DVC will be used for source control of code, data, and artifacts*
3. Are there constraints or considerations for data storage?	3. *The AtriumDB API will be leveraged to provide access to underlying physiological data. Project specific data requirements will be met through the existing Clinical Deep-Learning Infrastructure.*
4. Are there strict ethical or legal contraindications to the	4. *The project was approved by the local research ethics board with attention paid to how consent would be obtained (e.g., implied vs expressed), power dynamics, privacy, and protection of persons. The model's development will include attention*

This is a provisional file, not the final typeset article

proposed functionality of the model?	*to usability and staff acceptance/endorsement, performance measurement that includes attention to protected characteristics and strategies to redress potential biases, and considerations for post-integration socialization and communication with patients and families. The model itself is designed to identify a clinically relevant problem that can result in a demonstrable improvement to care in the unit, and ideally prevent harmful outcomes to patients*

AtriumDB – physiological data storage solution used; *DVC* – Data Version Control; *GPU* – Graphic Processing Unit; *NP* – Nurse Practitioner; *RN* – Registered Nurse.

1 References

1. Hannun AY, Rajpurkar P, Haghpanahi M, Tison GH, Bourn C, Turakhia MP, et al. Cardiologist-level arrhythmia detection and classification in ambulatory electrocardiograms using a deep neural network. Nat Med [Internet]. 2019 Jan 7;25(1):65–9. Available from: http://www.nature.com/articles/s41591-018-0268-3

2. Goodfellow SD, Goodwin A, Greer R, Laussen PC, Mazwi M, Eytan D. Towards Understanding ECG Rhythm Classification Using Convolutional Neural Networks and Attention Mappings. In: Machine Learning for Healthcare Conference. 2018. p. 83–101.

3. Goodfellow SD, Goodwin A, Greer R, Laussen PC, Mazwi M, Eytan D. Atrial fibrillation classification using step-by-step machine learning. Biomed Phys Eng Express [Internet]. 2018 May 8;4(4):045005. Available from: https://iopscience.iop.org/article/10.1088/2057-1976/aabef4

4. Goodfellow SD, Shubin D, Greer RW, Nagaraj S, McLean C, Dixon W, et al. Rhythm Classification of 12-Lead ECGs Using Deep Neural Networks and Class-Activation Maps for Improved Explainability. Comput Cardiol (2010). 2020;2020-Septe.

5. Goodwin AJ, Eytan D, Greer RW, Mazwi M, Thommandram A, Goodfellow SD, et al. A practical approach to storage and retrieval of high-frequency physiological signals. Physiol Meas [Internet]. 2020 Apr 20;41(3):035008. Available from: https://iopscience.iop.org/article/10.1088/1361-6579/ab7cb5
