# Supplementary material for: An integration engineering framework for machine learning in healthcare
Source: Front Digit Health. 2022 Aug 4;4:932411. doi: 10.3389/fdgth.2022.932411 (PMC9386122; doi:10.3389/fdgth.2022.932411)
Supplement: Supplementary file 2 [file Table_2.pdf]

Supplementary Materials

Supplementary Table 2 | Practical application of the proposed framework during the ***Preparation*** phase

Framework	Arrhythmia detection
**Technical System**	
1. Collect and prepare data for training and evaluation. Is data consistently, accurately, and reliably available for training, validation, and live function when deployed?	*1. To generate a mechanism to test the existing pipeline of data for model development as well as to generate additional arrhythmia labels based on the physiological waveforms in AtriumDB*(1)*, a waveform label application was developed that streamed retrospective waveform data to a user interface that was used by domain experts in the research team to generate labels and to evaluate the quality of the data overall. The success of this approach validated the adequacy of the existing pipeline and accuracy of the data for further training, validation, and model deployment.*
2. Does the retrospective training set have identical properties to the live data feed for predictions?	*2. Data in AtriumDB is stored verbatim from what it ingests from the real-time data feed, conserving all the properties that the model would see when itself receiving real-time data.*
3. Perform preliminary data analysis on real data. Is the data systematically biased and if so, can the bias be eliminated?	*3. Preliminary analysis on the data shows that it is possible to detect Junctional Ectopic Tachycardia using the proposed techniques. Further finetuning will help increase the accuracy of the model. The data is not believed to be biased as all patients in the Cardiac Intensive Care Unit are monitored similarly and based on their acuity as previously described in unit policies and protocols.*
4. Analyze user requirements in relation to potential model capabilities	*4. User requirements (a-e) based on the needs assessment that was conducted (described next) were deemed to be achievable given the current Machine Learning and software technology. Requirements f and g will be further investigated.*
**Human**	
1. Complete a formal needs assessment to define user requirements	*1. Needs assessment revealed the following set of user requirements: (a) accurate and live detection of Junctional Ectopic Tachycardia based on telemetry, (b) timely notification of relevant users (intensivist on call, medical trainee or NP as well as the RN responsible for the patient, (c) discreate notification without alarming patient and their parents as well as others in a shared room, (d) an explanation to show why the model suspects Junctional Ectopic Tachycardia, (e) a series of next steps to confirm diagnosis and management, (f) a feedback mechanism to correct the model when deemed incorrect by the clinical experts, and (g) model input into the current Electronic Health Record.*
2. Identify clinical and Artificial Intelligence related knowledge gaps	*2. Clinical gaps: Electrocardiogram based diagnosis of Junctional Ectopic Tachycardia and appropriate interventions to treat it were gaps among novice medical trainees, NPs, and RNs. Artificial Intelligence gaps: basic knowledge of Machine Learning techniques, probability-based model output, measures of accuracy, precision, and recall and their implications for*

	a prediction, expectation for performance degeneration, and effect of data quality on model performance were identified among all users.
3. Propose workflow and model use scenarios	*3. Multiple potential workflows were proposed including workflows that involved an automatic notification and diagnostic ordering through the existing Electronic Health Record, notification through a separate mobile application, as well as bedside notification with RN mediated activation of subsequent notifications. These workflows along with possible user interfaces were prototyped for usability testing in the next phase. Use scenarios generalize to that of a post cardiac surgery patient who is at risk of Junctional Ectopic Tachycardia.*
4. Determine need for explainability and actionability	*4. A need for an explanation for the model's output was identified in the needs assessment as well as a need for actionable output from the model guiding the next steps to follow once Junctional Ectopic Tachycardia is detected. Both were incorporated into the user requirements for the technical system.*
5. Evaluate feasibility and availability	*5. The proposed mechanism of generating labeled datasets using existing Electrocardiograms from the MUSE® database and AtriumDB was deemed to be feasible given the expertise of the clinicians within the study team. The appropriate model development expertise is readily available within the study group without the need to look outside the group for additional assistance. Designing and conducting summative and formative testing (described in the next phase) as well as silent trials, simulations, and prospective studies with broader representation from the stakeholder group and in the actual and simulated clinical space are feasible in the institution and through the research team as well.*
**Environment**	
1. What is the infrastructure needs in the clinical environment?	*1. Existing dedicated displays at each bedspace can be used to display the output of the Junctional Ectopic Tachycardia model. The displays are capable of displaying any web-based system. There is no current software system for displaying the output at the bedside, which represents the largest need from a clinical perspective. Data collection and model processing infrastructure are already in place and provide the necessary levels of reliability to responsibly deploy a system to the bedside.*
2. What are the limitations in existing privacy, security, and ethical considerations as it relates to the data and the proposed model?	*2. Need to develop clinical guidance to inform how the model's output should be utilized in clinical practice to tailor use to the specific parameters of our model's performance. Need to identify any ethical concerns pertaining to how representative the data may be compared to the population of patients cared for at the institution, the implications of underrepresentation on model outputs and clinical practice, and issues relating to algorithmic bias as a function of health inequities. Additionally, there is a need to identify potential concerns pertaining to clinician approved automatic ordering of diagnostic tests and interventions. Security and privacy concerns will also need to be investigated and addressed given the notification capabilities that are part of the user requirements.*
3. What are the requirements for the incorporation of such a model into the clinical space based on existing regulatory bodies?	*3. Food and Drug Administration requirements for clinical Machine Learning models were reviewed for this project. In addition, currently approved practices for model development as put forth by* (2) *and* (3) *are to be utilized in the development phase. As long as we follow these quality measures and complete a clinical trial, obtaining relevant approvals from regulatory bodies such as the Food and Drug Administration will be possible*

This is a provisional file, not the final typeset article

4. Is the technical system considered safety critical? If so, what are the appropriate fallback systems to ensure its consistent operation?	*4. The Junctional Ectopic Tachycardia model on its own is not considered safety critical and no backup systems will be considered for this model. However, the arrhythmia detection model as a whole will be considered safety critical and appropriate fall back systems will be considered for the broader system when available.*

AtriumDB – physiological data storage solution used; *MUSE* – Electronic repository for electrocardiograms; *NP* – Nurse Practitioner; *RN* – Registered Nurse.

1 References

1. Goodwin AJ, Eytan D, Greer RW, Mazwi M, Thommandram A, Goodfellow SD, et al. A practical approach to storage and retrieval of high-frequency physiological signals. Physiol Meas [Internet]. 2020 Apr 20;41(3):035008. Available from: https://iopscience.iop.org/article/10.1088/1361-6579/ab7cb5

2. Cruz Rivera S, Liu X, Chan AW, Denniston AK, Calvert MJ, Darzi A, et al. Guidelines for clinical trial protocols for interventions involving artificial intelligence: the SPIRIT-AI extension. Nat Med. 2020;26(9):1351–63.

3. de Hond AAH, Leeuwenberg AM, Hooft L, Kant IMJ, Nijman SWJ, van Os HJA, et al. Guidelines and quality criteria for artificial intelligence-based prediction models in healthcare: a scoping review. npj Digit Med. 2022;5(1).
