# Supplementary material for: An integration engineering framework for machine learning in healthcare
Source: Front Digit Health. 2022 Aug 4;4:932411. doi: 10.3389/fdgth.2022.932411 (PMC9386122; doi:10.3389/fdgth.2022.932411)
Supplement: Supplementary file 3 [file Table_3.pdf]

Supplementary Materials

Supplementary Table 3 | Practical application of the proposed framework during the ***Development*** phase

Framework	Arrhythmia detection
**Technical System**	
1. What is the process of developing the Machine Learning-software and its appropriate explanation?	*1. Junctional Ectopic Tachycardia detection model and its explanation will be developed iteratively based on user requirements previously identified and any technical challenges. The model and its explanation will also be tested with representative stakeholders at each iteration to determine the adequacy of the model's performance. Development and refinement will be done based on feedback from the human centered design process described next as well as the performance matrices used to evaluate the model and its explanation.*
2. What statistical tests are relevant for the model and how are they tuned in relation to user requirements?	*2. The current performance matrices of the Junctional Ectopic Tachycardia model are precision and recall (sensitivity). With each iteration of the model a new array of performance matrices will be generated and presented to the potential users to determine the adequacy of the model's performance.*
3. How is the model evaluated to ensure robustness and validity?	*3. The Junctional Ectopic Tachycardia model will be tested on a variety of datasets selected from the data in AtriumDB*(1). *The data sets will be realistic and matching what the model will see at deployment. To allow for a variety of testing, the datasets are of different sizes and some will have the either one or both of the CVP and arterial waveform missing to ensure model performance is primarily on the Electrocardiogram waveform.*
4. How is clinical validation achieved?	*4. A clinical validation study is designed to evaluate the clinical validity of the model against the performance of the clinicians including Electrophysiology specialists, Cardiac Intensive Care Unit intensivists, medical trainees, NPs, and RNs. These data will also be made available to users as part of the model's performance measures. This is a prospective longitudinal study which will be conducted with the finalized model and in a silent trial design such that the model would have no impact on patient management.*
**Human**	
1. What is the existing workflow and its challenges/limitations? How will the technical system fit into the existing workflow? How is the proposed fit evaluated?	*1. A task analysis was conducted at first to map the current workflow as it relates to the detection of Junctional Ectopic Tachycardia in a patient and the subsequent diagnostic investigations and interventions used to manage it. Based on this, a focused group of user representatives will be created who will identify areas of inefficiency and error in the existing workflow and propose how and where the model will be incorporated within the current workflow to minimize error and increase efficiency in the process. Consensus will be used to identify a few workflows which will be tested through low fidelity simulations with additional user representatives to determine the preferred workflow. This workflow will be tested in high fidelity simulation testing and modified as needed.*

2. How is the appropriate user interface designed for the technical system?	*2. User Interface for the Junctional Ectopic Tachycardia detection model will be designed using a human centered design approach focusing on the diagnosis of Junctional Ectopic Tachycardia and subsequent recommended steps. All standards for User Interface design will also be recognized and included in the design. The initial versions of this User Interface will be designed through focus groups of representative users as paper-based graphics and then digital mockups. The finalized proposed User Interface will be used for higher fidelity testing of the technical system during simulations and silent trials. Should further modifications to the User Interface be required following these tests, they will be incorporated into the final design.*
3. How are user-based acceptable performance matrices for the model determined?	*3. During low fidelity testing of proposed workflows and User Interface, as well as during higher fidelity testing in simulation, an acceptable range of model performance will be sought to guide model development overall. This will be achieved through posing a variety of performances in different mock scenarios and assessing the decision making of clinicians using a cognitive task analysis study to determine the optimal desired performance.*
4. How are the proposed workflow, model, user interface, and policies proposed for the model evaluated with the intended users and in the intended space? How are the cognitive impacts (if any) of the model on users evaluated?	*4. Multiple simulation studies of increasing fidelity with realistic scenarios will be conducted with representatives from all user groups. These simulations will be conducted at three stages, after desired model performance is achieved, after the desired model explanation is developed, and at the culmination of the development. The purpose of these simulations is to evaluate the proposed workflow, the User Interface and model performance, as well as the policies that are considered to guide the use of the model in the clinical space. All relevant feedback will be analyzed and used for further refinement before deployment. These simulations will also provide a platform through which cognitive load studies will be conducted using a variety of techniques such as eye tracking and retrospective cognitive task analysis with recorded simulations.*
5. What are some of the costs and impacts of the problem in the current state?	*5. Baseline assessment of current state will be conducted while the model is being developed. This includes understanding the current time to diagnosis of Junctional Ectopic Tachycardia, time to obtaining a 12 lead Electrocardiogram and atrial wire study to confirm the diagnosis from the preliminary diagnosis made based on telemetry, time to implementation of appropriate therapy, and time to detection of Junctional Ectopic Tachycardia resolution. In addition, number of consults to Electrophysiology and diagnoses confirmations with the Cardiac Intensive Care Unit intensivist will be calculated. These measures will be repeated after model deployment to evaluate the clinical impact of the model.*
6. How will the previously identified knowledge gaps be addressed?	*6. Educational sessions on Junctional Ectopic Tachycardia diagnosis and management as well as the principles of Machine Learning models are being developed to bridge the previously identified knowledge gaps among all users. Separate educational sessions will be developed to introduce the model, its functionality and limitations, as well as the associated workflow and policies.*
**Environment**	
1. What infrastructure is put in place to support a clinical model in a secure and reliable manner?	*1. The model will be deployed on the Clinical Deep Learning Infrastructure. This infrastructure is tolerant of failure and in the event of such a failure, will be automatically re-deployed to another server. This infrastructure is maintained as an isolated production environment with very tight access control. Additionally, model data both in-flight and persisted to disk is encrypted.*

This is a provisional file, not the final typeset article

2. What backup strategies are put in place in case the model is offline or the infrastructure supporting the model fails?	*2. In the event of a failure of the model, data can be re-retrieved directly from AtriumDB. In the event of a failure of the underlying infrastructure, there are automated backups of data that occur every hour and saved for a period of 1 year. The model itself is persisted in a version control system, which itself is backed up. Code is maintained on an enterprise Github account, which is protected under an SLA.*
3. How is the network, the infrastructure, and the fall back systems tested for stability and performance?	*3. Both logs and performance metrics of the underlying infrastructure are collected continuously on a central monitoring system. This system is leveraged to provide performance reports in the form of dashboards. Examples of collected metrics are network throughput, latency, CPU usage, memory usage, etc. In addition to dashboards, the system provides automated alerts from the metrics and notifies system / operational administrators with automated text messages in the event of failures or performance anomalies.*
4. How are model failure mechanisms and errors identified and addressed?	*4. Model failures because of underlying infrastructure troubles are identified by the monitoring system mentioned in the preceding section and responded to by operational administrators. Failures of the model pertaining to model performance, etc., are detected within the model and responded to currently by the owning data scientists. We plan to transition this to a central data science operational capacity.*
5. What privacy, use, regulations, and ethical policies and guidelines are needed for the model?	*5. Policies that would guide the use of the model, including a clear outline of Machine Learning medical directives, clinician responsibilities, and expectations will be developed. Given that the current patient monitors display alarms to notify clinicians of possible arrhythmias, we do not anticipate a need to change privacy policies associated to how the model output is displayed at the bedside but privacy considerations pertaining to how the output is communicated to the appropriate clinicians will need to be carefully determined. These guidelines will be evaluated during the final simulation testing.*

AtriumDB – physiological data storage solution used; *CPU* – Central Processing Unit; *NP* – Nurse Practitioner; *RN* – Registered Nurse; *SLA* – Service Level Agreement

1 References

1. Goodwin AJ, Eytan D, Greer RW, Mazwi M, Thommandram A, Goodfellow SD, et al. A practical approach to storage and retrieval of high-frequency physiological signals. Physiol Meas [Internet]. 2020 Apr 20;41(3):035008. Available from: https://iopscience.iop.org/article/10.1088/1361-6579/ab7cb5
