# Supplementary material for: An integration engineering framework for machine learning in healthcare
Source: Front Digit Health. 2022 Aug 4;4:932411. doi: 10.3389/fdgth.2022.932411 (PMC9386122; doi:10.3389/fdgth.2022.932411)
Supplement: Supplementary file 4 [file Table_4.pdf]

Supplementary Materials

Supplementary Table 4 | Practical application of the proposed framework during the ***Integration*** phase

Framework	Arrhythmia detection
**Technical System**	
1. What is the prospective clinical evaluation: randomized, controlled, etc.?	*1. A prospective randomized control trial is designed for this model to evaluate its clinical impact prospectively. The model is set to run in randomized bed spaces after deployment and its impact on patient care as determined by time to recognition, diagnosis confirmation, intervention, detection of resolution, and missed diagnoses due to false negatives, as well as workflow efficiencies, clinician, and patient satisfaction. Some of these measures will be influenced by other features of the model such as its false positive rate. Other trial structures that will be considered include randomization by time period (cluster) and cross over. Performance measures will be defined meticulously prior to the trial according to user expectations and power estimations.*
2. How is the model optimized and refined after deployment?	*2. Should there be any need to refine the technical system based on the broader system integration and user feedback, these modifications will be evaluated and ensued as appropriate.*
3. What mechanisms are used to monitor the system to ensure its performance is maintained?	*3. We monitor three types of metrics: System Metrics: Latency, Server Load, Throughput, Memory and Compute Utilization; Input Metrics: Min, max, mean Electrocardiogram amplitude, R-peaks, central frequency, missing data, heart rate; Output Metrics: model outputs a null value, and any additional feedback from the clinicians*
4. What mechanisms are there to monitor and track problems within the system?	*4. We will have a dashboard that will display all the information mentioned above. We could also set thresholds to flag any deviations from "normal".*
5. How are algorithmic biases monitored for and rectified?	*5. During the prospective clinical trial, additional data, such as patient nationality and other protected characteristics, will be collected and utilized to evaluate model outputs for potential bias in this regard.*
**Human**	
1. How are knowledge translation plans executed prior to model deployment?	*1. Knowledge translation sessions pertaining to the principles of Junctional Ectopic Tachycardia diagnosis and management as well as Machine Learning in general and the specific sessions pertaining to the Junctional Ectopic Tachycardia model will be completed prior to the deployment of the model. By the end of these sessions, it is expected that the users would be familiar with how the proposed model will be part of their current workflow and how its output should be critically evaluated before proceeding with its recommendations in keeping with the set Machine Learning directives, policies, and guidelines.*
2. What are just-in-time strategies to support users early in deployment?	*2. During the integration phase, experts in the technical system who are recruited from the study team and users who participated in the design process will be available to help users and answer any questions and concerns pertaining to the system.*

3. How is ongoing feedback regarding workflow and User Interface obtained during the integration phase?	*3. Any feedback that could further improve the system, including the policies surrounding the system will be taken into consideration to further refine and optimize the model for its intended clinical space. As part of this feedback, we will also seek feedback on issues such as alarm fatigue and communication challenges.*
4. What prospective cost analysis, efficiency considerations, etc. are considered?	*4. A prospective cost analysis as described in the prospective trial will be ensued when the model is deployed in the unit overall and compared to the preliminary cost analysis that was done during the previous phase.*
**Environment**	
1. How are new/modified policies and rules of engagement as it relates to model use implemented?	*1. Developed policies will be made live within the institution and made part of the routine operations. The existing mechanisms of cascading new changes to such policies will be used to inform users of these new policies. This is in addition to the knowledge translation sessions that are conducted prior to deployment.*
2. What is the impact of model on the broader system of systems?	*2. We do not believe this evaluation is necessary given that the model is locally deployed within the Cardiac Intensive Care Unit only.*

This is a provisional file, not the final typeset article
